# Tolerant and despotic macaques show divergent temperament but similar theory of mind

**DOI:** 10.1098/rstb.2024.0121

**Published:** 2025-06-26

**Authors:** Natalie Schwob, Rosemary Bettle, Megan Mulhinch, Zarin P. Machanda, Alexandra G. Rosati

**Affiliations:** ^1^ Department of Psychology, University of Michigan, Ann Arbor, MI 48109, USA; ^2^ Department of Psychology, Bucknell University, Lewisburg, PA 17837, USA; ^3^ Program in Animal Behavior, Bucknell University, Lewisburg, PA 17837, USA; ^4^ Department of Psychology, University of California San Diego, La Jolla, CA 92093, USA; ^5^ Departments of Anthropology and Biology, Tufts University, Medford, MA 02155, USA; ^6^ Department of Anthropology, University of Michigan, Ann Arbor, MI 48109, USA

**Keywords:** social cognition, theory of mind, primates, comparative cognition

## Abstract

The social intelligence hypothesis proposes that the demands of social life shape the evolution of cognition, but different aspects of social interactions may be relevant. To test how competitive versus cooperative interactions shape social cognition, we assessed multiple metrics of social cognition in Barbary macaques (*Macaca sylvanus*, *n* = 40) and rhesus macaques (*Macaca mulatta*, *n* = 60). These closely related species have similar social organization, but diverge in social styles: Barbary macaques are more tolerant, whereas rhesus macaques are more despotic. Monkeys completed a battery of experimental tasks measuring *gaze-following* (co-orienting with others), *knowledge attribution* (representing others’ underlying knowledge states), *goal attribution* (interpreting others’ actions in terms of underlying intentional goals) and *temperament* (boldness in response to exploring novelty). While the rhesus macaques were more willing to approach a novel object than were Barbary macaques, both species showed similar success in each social task. However, individual Barbary macaques were more likely to show greater overall proficiency across all social measures combined than were individual rhesus monkeys. Overall, these results indicate that similar social cognitive capacities may evolve in distinct social contexts, and suggest socio-cognitive skills may be relevant for both competitive and cooperative interactions in primates.

This article is part of the Theo Murphy meeting issue ‘Selection shapes diverse animal minds’.

## 1. Introduction

An influential hypothesis for the evolution of cognition suggests it stems from the demands of animals’ social lives [1,2]. For example, many primate species live in fairly large social groups with differentiated social relationships, and primates in general have long been noted to have a variety of sophisticated cognitive abilities [3,4]. However, some aspects of this ‘social intelligence hypothesis’ have been difficult to test. One important line of evidence in support of this view comes from comparisons of brain size and other neuroanatomical features. Some of this work shows that indices of social complexity, like group size, can be correlated with brain metrics (such as neocortex size), but other work has revealed conflicting results [5–7]. More generally, these brain measures may be only a rough proxy for more specific features of cognition [8,9]. Another line of work has tested the social intelligence hypothesis by comparing how actual cognitive performance relates to sociality, either within or between species. While some studies have found support for this link [1,10–13], others have not [14–17]. Overall, this indicates that the relationship between animals’ social lives and their cognitive abilities is still unclear.

One crucial consideration for testing the social intelligence hypothesis concerns the specific cognitive skills in question. Some studies indicate that components of social complexity—such as larger groups, more distinct relationships, or patterns of social interactions—may lead to a general shift in cognitive skills spanning domains including memory and executive function that impact a wide range of behaviours [10,18]. In contrast, other proposals focus on social cognitive skills that might be more specifically used to navigate social interactions [19–21]. For example, ring-tailed lemurs (*Lemur catta*) are a species of strepsirrhine primate that live in relatively large social groups with non-kin and dominance hierarchies unlike many other lemur species, and they are relatively more sensitive to social cues such as gaze and perception, but do not outperform other closely related lemur species on tasks assessing inhibitory control or spatial cognition [13,15,21,22]. More generally, larger-scale phylogenetic comparisons have produced divergent findings that depend in part on the skill in question: whether a particular cognitive skill covaries more with species’ social structures versus their dietary niche can depend on whether social cognition, social learning, or measures of inhibitory control are assessed [15,17,23,24]. Given that different aspects of cognition may evolve in a mosaic pattern [25,26] such that different skills can change independently in some cases, it is important to consider that social complexity may impact some domains of thought but not others across different species.

A second important consideration for testing the social intelligence hypothesis concerns how to appropriately index social complexity in animals [27–29]. Different proposals have highlighted several potential aspects of social life that might be relevant for cognitive evolution. To date, much work has focused on testing the importance of larger social group size, which may require cognitive capacities for tracking relationships with multiple social partners [10,11,13,30]. However, more specific aspects of social behaviour may also be relevant. For example, competitive social contexts have been proposed to be relevant for social cognition in several species, including primates, as robust social cognition may allow individuals to infer others' perspectives, knowledge and motives to better outmanoeuvre conspecifics [2,31–33]. Indeed, many animal studies assessing capacities for theory of mind, or attribution of subjective mental states to others, have implemented competitive contexts [34–36]. Conversely, some social cognitive skills have also been proposed to facilitate cooperative interactions [35,37,38]. For example, more tolerant species are more attentive to others’ gaze than more competitive species [19,39,40]. As such, it is important to understand how different social milieus may shape cognition.

In the current work, we tested how social competition versus tolerance shapes social cognition by comparing components of theory of mind and related social cognitive skills in Barbary (*Macaca sylvanus*) and rhesus (*Macaca mulatta*) macaques. The macaque genus offers a unique opportunity to understand the social cognitive evolution, as this evolutionary radiation produced multiple macaque species that are closely related and share several relevant socioecological features, such as overall social organization, but vary widely in social tolerance. Barbary and rhesus macaques specifically have high genetic relatedness, similar body and brain size, and live in multi-male, multi-female groups of similar size with similar patterns of female philopatry, matrilineal dominance hierarchies, and promiscuous mating [41,42]. Despite these overall similarities in social organization, these species show major differences in their social tolerance and patterns of related social behaviours like aggression and affiliation. On one end of the spectrum, rhesus macaques are highly despotic, with steep dominance hierarchies, more intense aggression and formal submission signals. Conversely, more tolerant species like Barbary macaques exhibit a flatter dominance hierarchy, more affiliative interactions, and more coalitions [43,44]. The differences in the social styles of these species, therefore, provide a ‘natural experiment’ to compare their cognitive skills, and dissect how different aspects of cognition may be linked to the two species’ patterns of sociality.

The current study, therefore, compared rhesus and Barbary macaques across multiple metrics of social cognition. We focused on three social tasks related to theory of mind that have been previously characterized in primates with relevant experimental tasks. First, we compared these species’ capacities for *gaze-following*, or co-orienting with another individual. Gaze-following is a foundational social cognitive skill for both humans and other primates, and is a necessary pre-condition for inferences about others’ visual perspective. While many primates show basic co-orienting responses, and this behaviour may depend on a variety of mechanisms [45,46], prior work shows that both of these macaque species specifically reason about others’ line of sight when gaze-following, as opposed to exhibiting more reflexive or automatic behaviours [47,48]. Second, we compared their skills for *knowledge attribution*, or inferring how others’ perception of an event shapes their current knowledge. Differentiating others’ knowledge from ignorance is a key theory-of-mind skill in humans and other animals, and there is similarly evidence that both of these species can make such inferences [32,49,50]. Finally, we compared *goal attribution*, or interpreting others’ behaviours in terms of underlying goals and intention states. Goal attribution is similarly a key component of theory of mind, and requires inferences about the underlying mental states impacting others’ behaviour as opposed to only tracking visible aspects of others’ physical movements. There is evidence that a variety of primates, including macaques, interpret others’ behaviours in this fashion [31,51,52]. We did not test for false belief attribution, a benchmark test for theory of mind in humans, as there is currently no evidence that macaques show this skill [32,50,53]. Importantly, all of these social cognitive capacities have been variously proposed to play a key role in both competitive and cooperative interactions in animals [34,54–56]. For instance, individuals may attend to where others are looking, what they know, and what they want in order to exploit this knowledge and then outwit groupmates when competing with them to access resources. Conversely, the same skill set could allow individuals to better infer others’ intentions and knowledge and therefore facilitate more efficacious cooperative interactions, such as by signalling their interest in grooming or group cooperation. The fact that these social cognitive skills could potentially be used to serve either cooperative or competitive outcomes therefore sets up an informative comparison of these macaque species.

In the current study, we therefore experimentally measured social cognition in a sample of 100 semi-free-ranging monkeys living in naturalistic environments and interacting in self-organized conspecific social groups. We constructed a novel battery of tasks that assessed these three social cognitive metrics—*gaze-following*, *knowledge attribution* and *goal attribution.* We further characterized *temperament* in the same individuals by including a task assessing responses to novel objects, as temperament may mediate or constrain cognitive performance and is therefore commonly accounted for in animal studies [57,58]. Some views further propose that differences in self-regulation may impact social cognition more generally [59,60] and therefore may shape potential differences in the monkeys’ social responses. We tested both species using identical methods that allowed us to characterize individual differences in social cognition, using tasks that are validated for semi-free-ranging primates [40,49,53,61]. We specifically included tasks that involved a neutral social context (e.g. not overtly competitive or cooperative in nature) to contrast these species. This approach allowed us to compare these species in equivalent contexts, as well as examine patterns of covariation in cognition across individuals. This battery of tasks allowed us to contrast key proposals about the relationship between cognition and social characteristics. If cooperation drives the emergence of social cognition, then more tolerant Barbary macaques should outperform rhesus macaques in our indices of social cognition. Conversely, if competition is the main driver of social cognitive evolution, then despotic rhesus macaques should be more successful in these tasks. A final possibility is that social cognition is relevant in both agonistic and affiliative social interactions, albeit for different reasons [62], in which case these species may show similar performance. Finally, we examined patterns of species- and individual-level variation across the different tasks to assess inter-relationships and scaling of performance in social cognitive and temperament metrics.

## 2. Methods

### (a) Subjects

We tested 100 semi-free-ranging male macaques from two sites: 40 Barbary macaques living in the Trentham Monkey Forest, Stoke-on-Trent, UK (mean age 10.7 ±4 .8 years, range = 4.1, 23.1), and 60 rhesus macaques living at the Cayo Santiago Biological Field Station in Puerto Rico (mean age 10.0 ± 4.5 years, range = 4.6, 22.3). Monkeys in both populations live in naturally formed social groups, are provisioned, and are habituated to observers and cognitive experiments [40,48,49]. All monkeys were over 4 years of age to reduce potential age-related variation, and were all male to reduce potential variation in behaviour and cognitive performance due to sex (additional aspects of the larger project involved observations of social behaviour, such as aggression and grooming, where there are known major sex differences). In some cases, specific individuals could not be located for a particular task in this free-ranging context and were then not included in relevant analyses (see details below and electronic supplementary material).

### (b) Overview of approach

In this pre-registered study (Barbary data pre-registration: https://aspredicted.org/XIE_WPI; rhesus data pre-registration: https://aspredicted.org/4F5_8HY; species comparison pre-registration: https://aspredicted.org/53M_433), macaques completed a battery of four experimental tasks over the course of approximately three months while they semi-free-ranged in their social groups. Monkeys completed the tasks in a fixed order: gaze-following, knowledge attribution, goal attribution and then, finally, the boldness measure. Monkeys completed no more than one experimental task per day, typically with a break lasting three weeks between each task.

To test monkeys, experimenters carried apparatuses and searched for target subjects, using the same general methods of several prior studies at both sites [40,48,49]. Macaques were tested while they were seated calmly in an appropriate location to present the specific cognitive test. One experimenter (the demonstrator) stood or knelt approximately 2−3 m from the monkey, attracted the monkey’s attention and then performed the experimental demonstrations; a second experimenter video-recorded the monkey’s responses for later coding. Methods for tasks were generally identical across both sites (see electronic supplementary material for details). In this free-ranging context, monkeys approached for testing occasionally did not produce a scorable response (for example, because they were disrupted by another monkey) and then could not again be located for testing on that task. In these cases, that individual was excluded from relevant analyses. All 100 macaques completed the gaze-following task; 95 monkeys (36 Barbary, 59 rhesus) completed the knowledge attribution task; 97 monkeys (38 Barbary, 59 rhesus) completed the goal attribution task; and 99 monkeys (40 Barbary, 59 rhesus) completed the temperament task (see electronic supplementary material for details of individual exclusions by each task).

### (c) Specific task procedures

As the primary goal of this study was to assess individual variation in responses in the cognitive tasks, and the tasks used here can produce habituation-related declines over trials, we presented the stimuli in a fixed order in each task, following prior work [58,63,64]. The logic of this experimental design is that a fixed order is a more sensitive design to detect individual variation in responses without the confound of different condition orders. All tasks were videotaped by the second experimenter and coded from video.

**Figure 1.**
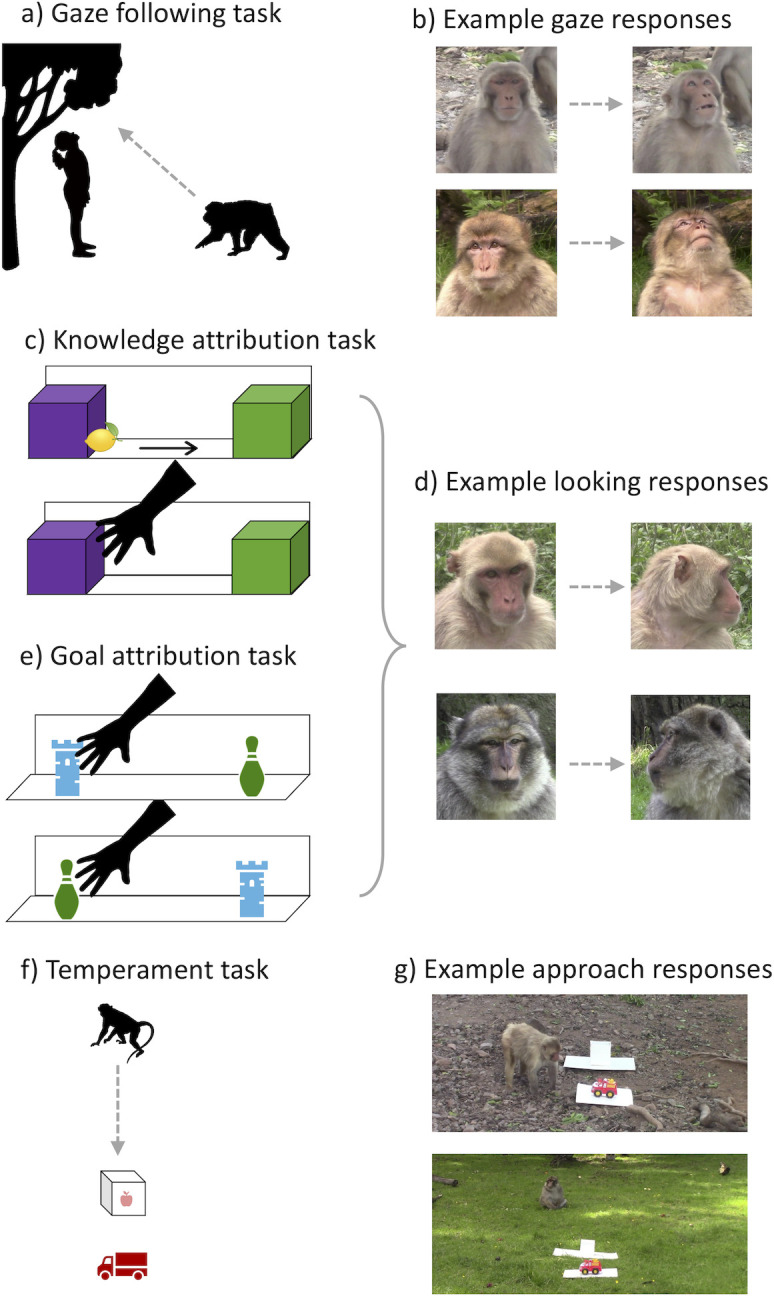
Methods for the cognitive battery. (*a*) Setup for the *gaze-following task*: a human demonstrator looked either up (test trials) or down (control trials). (*b*) Example monkey gaze-following responses: left panel shows monkey looking at the actor, and right panel shows upwards look for a rhesus macaque (top row) and a Barbary macaque (bottom row). (*c*) Setup for the *knowledge attribution task*: a human demonstrator watched a fruit move on an apparatus and reached either to where it went (expected outcome) or to an empty location (unexpected outcome). (*d*) Example monkey looking-time responses: left panel shows monkey looking to the apparatus, and right panel shows looking away from the apparatus for a rhesus macaque (top row) and a Barbary macaque (bottom row). (*e*) Setup for the *goal attribution task:* a human demonstrator first reached for one of two possible items, and then, when their locations were switched, either continued to reach for it in a new location (expected outcome) or performed the same reach on a new object (unexpected outcome). (*f*) Setup for the *temperament task*: a monkey could choose whether to approach a fake-baited food box and a novel object (a toy) placed on the ground. (*g*) Example monkey approach responses: a rhesus macaque approaching the novel object (top panel) and a Barbary macaque choosing not to approach (bottom panel).

In the *gaze-following* task, we used methods validated at both sites [40,47,48,61] to test macaques’ propensity to co-orient with a human demonstrator (see figure 1a,b; electronic supplementary material, figure S1 for photographs and video S1 for example demonstration and monkey responses). After attracting the monkey’s attention to her face, the experimenter either looked up (*test trials*) or down (*control trials*) while the second experimenter video-recorded the monkey for the subsequent 10 s. Monkeys completed up to four trials, alternating test trials with control trials in two pairs; they had to complete at least the first pair of trials to be included. If monkeys followed the demonstrator’s gaze, they should look up more often on test trials than control trials.

In the *knowledge attribution* task, we used methods validated with macaques [49,50,53] that were adapted from prior work with human infants [65], to assess if monkeys expect an agent to act in a way that is consistent with their knowledge. We used a violation-of-expectation looking-time method, a well established technique to assess cognition in human infants [66] and animals [67,68], which measures the time spent looking at an event as an index of an individual’s underlying cognitive processes. Monkeys completed a sequence of four trials as in prior implementations of the basic knowledge task [49,53], where they observed a human observing or reaching into an apparatus in different configurations (see figure 1c,d; electronic supplementary material, figure S2 for photographs and video S2 for example demonstration and monkey responses); monkeys had to complete all four trials (the first two habituation trials and final two test trials) to be included. On each trial, the experimenter first attracted the attention of the monkey to the apparatus, and the monkey then observed as the experimenter watched a fruit move across the stage into a box, and then she reached into one of two boxes. In the critical test trials, the demonstrator either reached into the box where she saw the fruit go (the *expected* outcome) or reached into the empty box (the *unexpected* outcome). We measured the time spent looking at the apparatus for the subsequent 10 s. If monkeys understand that others act in accordance with their knowledge, they should look longer at the unexpected event where the demonstrator’s reach is inconsistent with her visual perceptions.

For the *goal attribution task*, we adapted validated methods from human infants [69,70] and macaques [20] to assess if monkeys predict others to act in a way that is consistent with their goals, again using violation-of-expectation looking-time methods. Monkeys completed a sequence of four trials where they saw a demonstrator reaching for one of two possible objects (see figure 1d,e; electronic supplementary material, figure S3 for photographs and video S3 for example demonstration and monkey responses); monkeys had to complete all four trials (consisting of two pairs of a habituation trial follow by a matched test trial involving the same objects) to be included. On each trial, the experimenter first attracted the attention of the monkey to the apparatus, and then effortfully reached for one of two distinct objects on the stage, signalling her goal; in the paired test trial, the location of these same objects was then switched. In the *expected* outcome, she reached for the object she originally wanted (in the new location), whereas in the *unexpected* outcome, the demonstrator then reached for the other object (e.g. performing the identical action she had before, but now with a different goal object). We measured time spent looking at the apparatus for the subsequent 10 s. If monkeys predict others act in accordance with their underlying goals, they should look longer at the unexpected event where the demonstrator’s reach does not match her initial intentions.

Finally, in the *temperament task* assessing boldness, we utilized common methods used with primates [15,71,72] to assess the monkeys’ willingness to approach a novel object (see figure 1f,g; electronic supplementary material, figure S4 for photographs and video S4 for example demonstration and monkey responses). Monkeys completed a single trial where the experimenter attracted their attention and then placed a box apparently baited with food, and then placed a novel object (a toy) behind the box; the food box aimed to ensure that monkeys were willing to move towards the objects. We measured whether the monkey chose to approach and investigate the box and the novel object. Here, bolder monkeys should be more interested and faster to approach these items than shyer monkeys.

### (d) Coding and analyses

All videos were scored by two experienced, independent blind coders. For coding, individual trials were clipped from the longer video sessions and given random number codes so that coders were blind to condition and trial number in a task. Reliabilities were determined by calculating Cohen’s kappa (gaze and temperament tasks with categorical outcome variables) or Pearson’s *r* (knowledge and goals tasks with continuous outcome variables). The coders showed high reliability at or above 0.92 for all measures (see electronic supplementary material for all coding and reliability details).

All analyses were completed using R v. 4.2.3 [73]. We first analysed trial-by-trial performance on each individual task to compare species’ performance in each metric. In the social tasks, we used mixed models implemented in the lme4 package [74] with either a binomial or linear distribution as appropriate. We compared whether model fit was improved by inclusion of additional predictors of interest (e.g. condition and species) using likelihood ratio tests [75], and report *post hoc* comparisons with Tukey corrections as relevant. This approach entails comparing competing models to test hypotheses by determining whether the inclusion of these additional predictors sufficiently improves the fit of the data compared with a model without these predictors [75]. Here, our base models generally accounted for individual *identity* (to account for repeated trials), subject’s *age* and any experimental predictors like *trial number* as relevant for the task. We then compared this base model with a model that sequentially added *condition* (e.g. expected versus unexpected outcome), *species* (Barbary versus rhesus) and then a model with a *condition × species* interaction to account for any potential differences in the species’ condition response. We used a similar approach for the temperament data (comprising a single trial) using logistic regressions (see electronic supplementary material for details).

For some analyses, we further calculated a *difference score* to index an individual’s overall performance in the task using a single metric, given that each task consisted of multiple trials. Comparable difference scores are often used in studies of human infants and animals for both gaze-following and looking-time tasks [66,76–79], and are premised on the logic that the task understanding is reflected in greater looking to unexpected test events compared with expected control events while also accounting for baseline looking rates more generally; that is, positive scores indicate task comprehension based on the logic of the experimental design. Across the tasks we calculated the score by subtracting the relevant control condition performance from test condition performance (e.g. *test trial* looking up*— control trial* looking up in the gaze-following task; *unexpected test* looking time* — expected test* looking time in the knowledge task; we modified this looking-time score for the goals task to also account for matched habituation trial looking time, as monkeys showed longer baseline looking at one of the paired object sets compared with the other; see electronic supplementary material for details of this score’s calculation for each specific task). We then used this score to assess if temperament task response predicted social performance in linear regressions where we accounted for individual’s *age* and *species*, and then added *boldness* and *species × boldness* to assess if this improved fit. To validate the use of this score as a single metric of performance, we also compared difference scores across species to assess if this aligned with trial-by-trial analyses, both in specific tasks and for links to temperament.

We also used these scores to assess inter-relationships across the social tasks. This included bivariate correlations across social tasks, and a comparison of species’ integrative performance across tasks using a principal components analysis (PCA). Finally, we further used this difference score to index if individual monkeys ‘passed’ a given task in a binary fashion. As noted above, the logic of this difference score is that individuals with a positive value show performance consistent with task comprehension (e.g. the monkey looked relatively longer at the unexpected or test event). As such, these individuals were considered to have passed, allowing us to test interdependence in performance across the tasks. Specifically, we used this categorical pass/no-pass metric to assess a scalogram sequence of success, following prior work with human infants using the same general approach to understand task inter-relationships over development [80,81].

## 3. Results

### (a) Performance in social tasks

**Figure 2.**
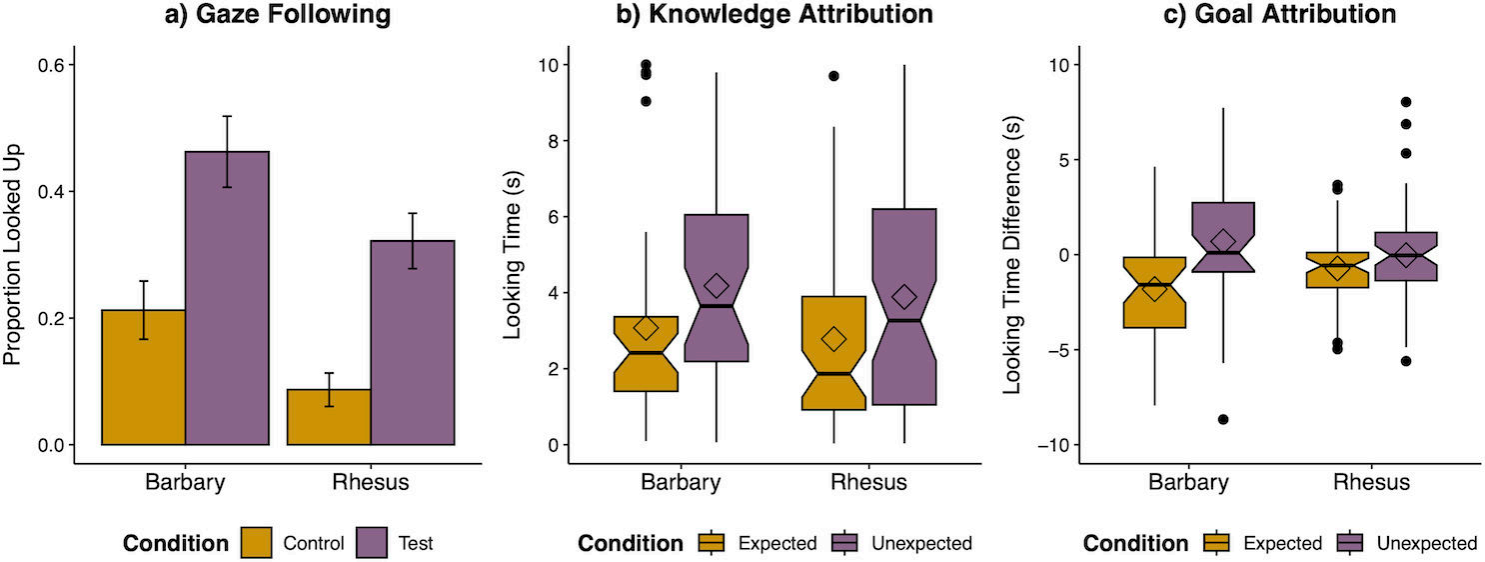
Social cognitive performance across species. (*a*) Proportion of monkeys that looked up in test versus baseline control trials in the *gaze-following* task. Successful monkeys should look up more often in test trials than in control trials. Error bars indicate s.e. (*b*) Looking times in expected versus unexpected trials in the *knowledge attribution* task. Monkeys should look longer at unexpected than expected test events. (*c*) Looking time change (habituation looking – test looking) for the expected and unexpected trials in the *goal attribution* task; relative change in the test trial compared with the paired habituation trial is depicted because monkeys differed in their initial interest in the different sets of objects used in each pair of trials. Monkeys should look longer at unexpected than expected test events, after accounting for this baseline difference. In boxplots, hinges indicate the lower and upper quartiles, the horizontal line represents the median, diamonds indicate the mean, and whiskers indicate the minimum and maximum range of data; outliers are plotted as individual points.

We first compared the two species’ propensity to follow gaze. On average, macaques looked up on 37.50 ± 3.65% (s.e.) of test trials, and 14.00 ± 2.76% of control trials (figure 2a). In models, inclusion of *condition* improved fit (*χ*² = 33.98, d.f. = 1, *p* < 0.001), indicating monkeys looked up more in test trials overall. Inclusion of *species* (*χ*² = 10.67, d.f. = 1, *p* < 0.001) also improved model fit: Barbary generally looked up more than rhesus. Finally, adding the *condition × species* interaction did not further improve fit (*χ*² = 0.64, d.f. = 2, *p* = 0.42), indicating similar responses to the trial types across species. Indeed, both species individually showed more looks in the test trials (see electronic supplementary material). An additional analysis of age-related shifts showed that Barbary macaques looked up more frequently than rhesus macaques in test trials specifically in old age (see electronic supplementary material for details), consistent with evidence of age-related shifts in gaze sensitivity in these species [40]. Finally, in examining the metrics of individual performance, there was no difference in their average difference scores in the task (*t* = −0.09, d.f. = 98, *p* = 0.93; Cohen’s *D* = 0.019) and a similar number of individuals ‘passed’ in both species (50.0% of the Barbary and 40.0% of the rhesus), aligning with the trial-by-trial analyses. Overall, this pattern of results shows that both species were proficient at following gaze; while there was evidence of greater responsivity to the gaze cues in the older Barbary macaques, the species’ performance was overall similar in this sample.

In the knowledge attribution task, macaques looked an average of 4.00 ± 2.85 (s.e.) s at the unexpected test trial, and 2.89 ± 2.52 s at the expected test trial (figure 2b). Inclusion of *condition* improved model fit (*χ*² = 13.15, d.f. = 1, *p* < 0.001): macaques looked longer when the actor’s reach did not align with their prior knowledge in unexpected trials than when it did in expected trials. However, neither the inclusion of *species* (*χ*² = 0.81, d.f. = 1, *p* = 0.37), nor a significant *condition* × *species* interaction (*χ*² = 0.81, d.f. = 2, *p* = 0.67], further improved fit. This indicates that both species showed a similar ability to predict that the demonstrator would act in accordance with her knowledge. Models of each species’ performance further showed that both species individually distinguished the conditions, and we did not find evidence for a shift in performance with age in either species (see electronic supplementary material for details). Finally, we found similar difference scores across both species (*t* = −0.008, d.f. = 93, *p* = 0.99; Cohen’s *D* = 0.002); 77.2% of individual Barbary macaques and 62.7% of rhesus macaques successfully passed the task by this metric, aligning with the trial-by-trial analyses. Overall, this shows that the two species also displayed similar ability to predict how others’ knowledge leads to action.

In the goal attribution task, macaques looked an average of 2.99 ± 2.62 s at the unexpected test trial, and 2.79 ± 2.57 s at the expected test trials. Initial checks showed that monkeys looked longer overall at trials involving the second set of objects (see electronic supplementary material), so here our primary analyses also accounted for this baseline looking to the object set in the matched habituation trial as a covariate in statistical analyses. When accounting for this baseline object preference, we found relative increases in looking to the unexpected test trial versus the expected test trial (figure 2c). Specifically, adding *condition* to our base model improved fit (*χ*² = 4.98, d.f. = 1, *p* = 0.026); macaques looked longer when the demonstrator reached for the new object in the unexpected trial than when she reacted for the goal object in the expected trial. However, neither the inclusion of *species* (*χ*² = 0.20, d.f. = 1, *p* = 0.65), nor the *condition × species* interaction (*χ*² = 1.63, d.f. = 2, *p* = 0.44) improved fit, indicating similar responses across species. Here, species-specific models did not find a condition effect in either individual species, but rather only as a group (see electronic supplementary material), and we did not find any shifts with age (see electronic supplementary material). However, we did find that the species varied in the overall performance metrics: Barbary showed higher difference scores, indexing task compression, than rhesus [*t* = 2.29, d.f. = 95, *p* = 0.024; Cohen’s *D* = 0.48], and accordingly, whereas 65.8% of Barbary passed by this metric, only 59.3% of rhesus macaques did so. While this provides some evidence of greater sensitivity to goal cues in Barbary macaques, it is important to note that the two species’ performance was similar in the full trial-by-trial analysis.

### (b) Temperament and relationship with social performance

**Figure 3.**
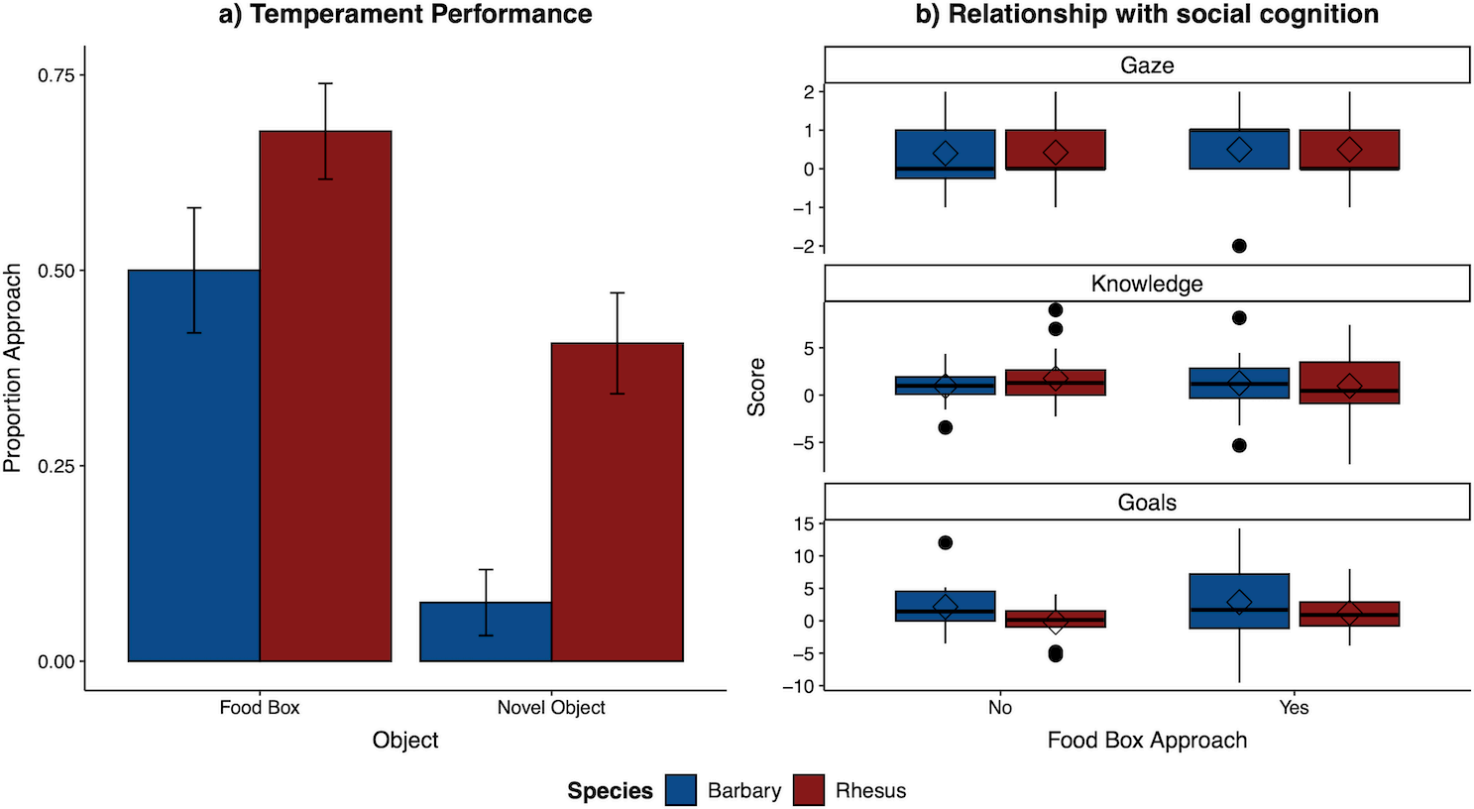
Boldness and social cognitive performance across species. (*a*) The proportion of monkeys in each species that approached the food box and the novel object in the *temperament task*. Error bars indicate s.e. (*b*) Variation in social task *difference scores* indexing individual’s overall performance in that task, relative to individual performance in the temperament task. Positive difference scores indicate response patterns concordant with task comprehension (e.g. looking up more in test than control trials in the gaze task, or relatively longer looking at the unexpected compared with the expected test trials in the knowledge and gaze attribution tasks). In boxplots, the horizontal line represents the median, diamonds indicate the mean and whiskers indicate the minimum and maximum range of data; outliers are plotted as individual points.

In our temperament task assessing boldness, 50.0% of Barbary and 67.8% of rhesus macaques approached the food box, whereas only 7.5% of Barbary versus 40.7% of rhesus macaques subsequently approached the novel object (figure 3a). For analyses of approaches to the food box, inclusion of *species* did not significantly improve model fit (*χ*² = 2.78, d.f. = 1, *p* = 0.096), and individuals that did approach did so at similar speeds in both species (*χ*² = 1.33, d.f. = 1, *p* = 0.846), indicating that that both species were similarly willing to move towards the testing setup and motivated by food rewards. However, analyses of approaches to the novel object specifically showed that inclusion of *specie*s improved model fit (*χ*² = 14.60, d.f. = 1, *p* < 0.001): rhesus macaques were more likely to approach than Barbary macaques. Given that only three Barbary macaques approached the novel object at all, we did not compare latency across species for this measure. Overall, these results show that while both species showed similar motivation to search the food resource, rhesus macaques were bolder in specifically exploring novel objects than were Barbary macaques.

We then examined whether boldness predicted performance across the three social tasks. To do so, we again indexed overall performance for each task with the difference scores, and assessed if that monkey’s temperament response predicted social performance either overall or by species. In fact, there was no improvement of model fit with the inclusion of boldness, or its interaction with species, for any of the social tasks (*gaze-following* task: *χ*² = 0.36, d.f. = 1, *p* = 0.497; *knowledge task*: *χ*² = 0.88, d.f. = 1, *p* = 0.743; *goals task*: *χ*² = 5.57, d.f. = 1, *p* = 0.537; see figure 3b and electronic supplementary material for full reporting of these comparisons). We also conducted additional checks of this result using the full trial-by-trial social data, rather than the individual difference scores; these analyses mirrored the primary analyses of each social task, and then also included boldness as a predictor (see electronic supplementary material for details). This check showed similar results, such that temperament did not predict social performance in any of the tasks. Overall, this indicates that the species differed in their boldness in terms of willingness to approach novel objects, but bolder versus shyer individuals did not perform differently on the social tasks at either the species or individual level.

### (c) Inter-relationships between social metrics

**Figure 4.**
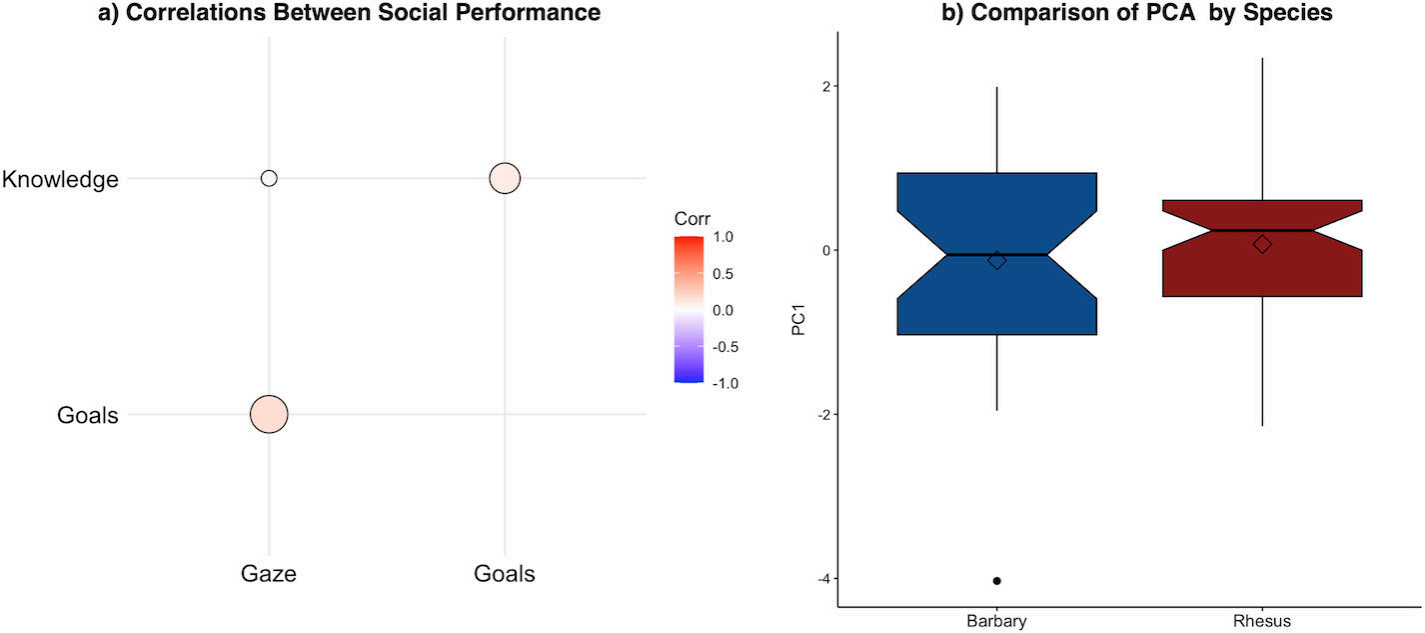
Relationships between social cognitive tasks and species comparison of overall performance. (*a*) Bivariate correlations between the difference scores in the social cognition tasks; tasks did not show significant shared variance. (*b*) Average summary scores for Dimension 1 extracted from the PCA; the species did not differ in this integrative measure of social performance. Hinges indicate the lower and upper quartile, the horizontal line represents the median, diamonds indicate the mean, and whiskers indicate the minimum and maximum range of data; outlier is plotted as an individual point.

We next examined the relationships across the different social cognitive measures. As an initial test, we tested for bivariate correlations between individual’s difference scores across the three social tasks (figure 4a). We found no relationship between any of these three measures (gaze versus knowledge: *r* = 0.03, *p* = 0.80; gaze versus goals: *r* = 0.16, *p* = 0.125; knowledge versus goals: *r* = 0.09, *p* = 0.372), indicating no clear shared variation across different tasks. This suggests that the three social tasks in fact measured distinct aspects of social cognition, without much overlapping variance in individual performance. We then conducted a PCA to extract summary scores of each individual’s responses (see electronic supplementary material for all details), allowing us to directly compare the two species on this integrative measure of social performance. While the PCA did not meet all criteria given the few tasks included (see electronic supplementary material for details), comparison of the single principal unrotated component indicated that the species did not differ by this measure either (*χ*² = 1.58, d.f. = 2, *p* = 0.664; figure 4b). Overall, this result aligns with the species comparisons of trial-by-trial performance in each task, indicating little variation by species.

We finally examined patterns of ‘passing’ performance across the two species. As noted previously, to do so, we dichotomized the difference score from each task such that individuals that showed positive scores (e.g. looking or gaze patterns across condition congruent with task comprehension), and then used this passing metric to assess the rate at which monkeys passed all three social cognition tasks. Whereas 35.29% of Barbary macaques passed all three tasks, only 11.86% of rhesus did. Comparisons of passing rate indicated that Barbary macaques were more likely to succeed across the board (*χ*² = 7.48, d.f. = 1, *p* = 0.006). This suggests that while the two species showed similar responses in individual tasks, Barbary macaques showed better performance when considering ‘success’ across all the tasks. We then used these passing scores to assess the scaling of performance across tasks, following an analytical technique used in studies of the development of social cognition to understand the sequence of comprehension across distinct theory-of-mind components [80,81]. In particular, human children show a stable pattern of progression through distinct social understandings (e.g. infants follow gaze before understanding false beliefs). Examining which theory-of-mind skills monkeys were more broadly proficient in and the patterning of skills can therefore pinpoint the links between different skills. In fact, we found that 67.74% of monkeys aligned with a pattern where they were proficient in knowledge attribution, followed by goal attribution, and finally, gaze-following was the skill the least number of monkeys ‘passed’ (table 1). That is, most individuals that succeeded at the gaze-following task also succeeded at the knowledge and goals tasks, whereas monkeys rarely succeeded at gaze-following if they did not also succeed at knowledge and goal attribution. Examining the species separately further showed that Barbary macaques most often passed all three tasks (35.29%) whereas rhesus most often just passed knowledge and goal attribution (25.42%), in line with the finding that Barbary macaques showed stronger overall performance.

**Table 1.** Scalogram patterns for success across the social tasks. A plus sign indicates macaques that ‘passed’ the task (according to their dichotomized difference score indexing relative performance in the test versus control conditions), whereas a minus sign indicates failing the task by this metric. The percentage indicates the proportion of macaques falling into each pattern category, followed by the total number of individuals in parentheses. Monkeys following pattern 4 passed all three tasks; those in pattern 3 passed both the knowledge and goal attribution tasks but not the gaze task, those in pattern 2 passed only the knowledge task; and those in pattern 1 failed all three tasks. Of the total sample, 93 macaques completed all three tasks; additional monkeys did not fall into the primary scaling pattern.

pattern	1	2	3	4
knowledge	−	+	+	+
goals	−	−	+	+
gaze	−	−	−	+
all	10.75% (10)	13.98% (13)	22.58% (21)	20.43% (19)
Barbary	14.71% (5)	17.65% (6)	17.65% (6)	35.29% (12)
rhesus	8.47% (5)	11.86% (7)	25.42% (15)	11.86% (7)

## 4. Discussion

Our results indicate that tolerant Barbary macaques and despotic rhesus macaques show similar patterns of social cognition despite major differences in their patterns of social behaviour and tolerance. We implemented three tasks targeting different components of primate social cognition using well validated methods for these semi-free-ranging populations. We found that both species were similarly successful at following another individual’s gaze direction, inferring another individual’s knowledge states based on what they had previously observed and interpreting another’s actions in terms of underlying goals—with no major differences in the species’ performance in any of these individual tasks. We also showed that rhesus monkeys were bolder than the Barbary macaques in terms of their willingness to approach and investigate a novel object, in line with differences in these species’ social style, but neither species-level nor individual-level variation in temperament related to social cognitive performance. Overall, our findings suggest that differences in social tolerance across macaques, and even in temperament, do not necessarily lead to differences in social cognition.

Our work bears on influential views concerning how sociality may shape the evolution of social cognition. Several proposals argue that social context may play a crucial role in the evolution of theory of mind, highlighting the influence of either cooperation or competition as especially important for the emergence of social cognition [2,38]. However, our results suggest that even species with divergent patterns of social interactions, such as affiliation and aggression, can show quite similar patterns of social cognitive performance. This suggests that these social cognitive skills may be broadly adaptive for primates living in sufficiently large or complex social groups, and that both tolerance and despotism may independently promote the evolution of these abilities for their respective social contexts. Importantly, while these species vary in their social tolerance, both Barbary and rhesus macaques live in social groups with similar social structures, group sizes, and organizations comprising large multi-male, multi-female groups with a variety of both kin and non-kin relationships [41,42,44]. As such, both species may use social cognitive skills to navigate their social landscapes, albeit potentially for different purposes. For example, rhesus macaques may use theory of mind to see what resources conspecifics are interested in to better outcompete others (e.g. another’s knowledge of a food resource), whereas Barbary macaques may use theory of mind to facilitate affiliative interactions (e.g. another’s intention to groom). In that sense, these divergent social structures may produce the same cognitive outcome.

We did find some subtle differences in the species’ performance that are consistent with the tolerant Barbary outperforming the despotic rhesus macaques. For example, although both Barbary and rhesus macaques successfully followed gaze, Barbary macaques showed higher rates of gaze-following specifically in old age than rhesus macaques, as in some prior work [40], which aligns with a larger body of evidence that tolerant species may be especially sensitive to gaze direction and eye contact [39,40]. We also saw that individual Barbary macaques showed more positive difference scores in the goal attribution task than rhesus, which is consistent with greater task comprehension, and Barbary macaques were also more likely to pass all three tasks overall. However, direct comparisons of Barbary and rhesus macaques’ trial-by-trial performance in each of the individual tasks show no significant differences, nor did they differ in the holistic comparison of performance, such as using PCA-derived values based on the task difference scores. Notably, while there is debate on how to measure social complexity in animals, recent work suggests that more tolerant macaques have more complex social groups than despotic species, contributing to the greater ‘degrees of freedom’ in their social interactions [29]. These findings suggesting subtle differences where the Barbary outperform the rhesus are in line with this possibility, and highlight the importance of accounting for not only performance on single social tasks, but also more global patterns of performance across multiple cognitive metrics.

These results from the social tasks should be interpreted with reference to several methodological considerations. First, we focused on components of social cognition that have been previously documented in nonhuman primates, but we did not test other aspects of human-like theory of mind, such as perspective-taking and false belief attribution. While macaques do not seem to engage in false belief reasoning [32,50,53], future work could assess measures of perspective-taking that have been validated in macaques. Second, we used tasks where monkeys made inferences about a human demonstrator for practical reasons, as it is difficult to control the behaviour of a conspecific in the way necessary for such experiments. However, it is important to note that prior work suggests that many primate species, including macaques, apply social skills like gaze-following to both humans and conspecifics in relevant contexts [40,61,82,83], and more generally, many demonstrations of social cognition in macaques have involved human actors [32,49,50,53,84,85]. Finally, all three tasks consisted of ‘neutral’ contexts, in the sense that the actor did not engage in either overt competition or cooperation with the monkey. This is in contrast to many other studies demonstrating evidence for theory of mind in primates involving competitive situations where animals make social inferences to out-compete others and acquire a contested food treat [13,36,86], or initially engage in positive communicative cues to signal cooperation [34,35]. None of our tasks involved such overtly competitive or cooperative social interactions. One important direction for future work would be to frame the same social problem as cooperative versus competitive to compare these species, as in some prior work with apes [87]. For example, Barbary macaques might outperform rhesus macaques in cooperative contexts, and conversely, rhesus might outperform Barbary in a more competitive regime. Nonetheless, it is important to stress that both species performed well in the current neutral implementation of these tasks.

These similarities in cognitive performance are also surprising given that the two species diverged in their boldness in the temperament task: more rhesus macaques approached the novel object, whereas few Barbary macaques did. Given the well documented differences in aggression between these macaque species [44], this difference in boldness is in line with theoretical distinctions between proactive and reactive temperament styles [88], proposing that proactive individuals are quicker to explore their environment and are more aggressive, and reactive individuals are less exploratory and less aggressive. This pattern is also in alignment with prior work with apes showing that more despotic chimpanzees are also more willing to approach novel objects and situations than are relatively tolerant bonobos [71]. While some proposals link temperamental characteristics to the emergence of social cognitive skills in humans and primates [60,89], we did not find any evidence that temperament was related to social cognition in the macaques. However, we note that our measure of temperament was a one-shot response to a single novel object, so future work could assess temperament and personality in these species in more depth. For example, it would be relevant to assess if they show consistent differences in responses to multiple novel stimuli to better characterize general boldness across individuals [15,71]. In addition, comparisons of temperament and personality across multiple distinct dimensions [90–92] could identify the more general structure of stable individual differences in these species.

Another novel aspect of our results concerns scaling different components of social cognition in these monkey species to examine the structure of their interdependence. In humans, components of a mature theory of mind develop in a consistent order such that later-emerging skills are dependent on the acquisition of earlier skills [93,94]; we adapted these analytical techniques from developmental psychology for the current work. In humans, the skills we tested in monkeys also emerge in a specific order: gaze-following is a skill that emerges within the first 3−6 months of life and scaffolds other capacities including perspective-taking [95,96]; interpreting others’ actions in terms of underlying goals develops by the end of the first year of life [70,97]; and skills like knowledge attribution tend to emerge later [81]. Using the structure of interdependence in adult macaques, however, we found a different pattern. Whereas most individuals demonstrated proficiency in the knowledge task, gaze-following was the most difficult for the monkeys. This apparently distinct sequence aligns with emerging evidence that humans and nonhumans might acquire some of the same social cognitive skills, but via different pathways. For example, in a developmental comparison of multiple cognitive tasks, nonhuman ape infants acquired many of the same skills as human children but did so in a different sequence [98]. Overall, this highlights the importance of examining not only the emergence of individual social cognitive skills in nonhuman animals, but also the patterns of their inter-relationships [99].

In sum, we compared two closely related species of macaques that vary in social tolerance on a novel battery of social cognitive and temperament tasks. Both species showed similar proficiency in multiple social cognitive components, despite differences in bold versus shy temperaments as well as more general differences in their social style and social behaviour. These results suggest that social cognition and theory of mind may be broadly important social skills for primates living in sufficiently complex social groups, such that these skills are advantageous across a wide variety of species. Thus, a crucial question for future work concerns the specific function of these skills in animals’ day-to-day lives. The current study sets the stage for future work empirically testing the relationships between individual variation in cognition and individual variation in patterns of social behaviour, such as affiliation and aggression, given that both study sites are home to large populations of monkeys living in naturalistic social groups with species-typical patterns of social behaviour. Examining how different social cognitive traits are linked to animals’ ability to navigate their social landscape is the next step for understanding the processes shaping the evolution of cognition.

## Data Availability

Data and analysis scripts are publicly available on the Dryad Data Repository at [100]. Supplementary videos documenting task procedures are available as part of the supplementary material [101].
